# *In vivo *trans-specific gene silencing in fungal cells by *in planta *expression of a double-stranded RNA

**DOI:** 10.1186/1741-7007-8-27

**Published:** 2010-03-31

**Authors:** Maria Laine P Tinoco, Bárbara BA Dias, Rebeca C Dall'Astta, João A Pamphile, Francisco JL Aragão

**Affiliations:** 1 Embrapa Recursos Genéticos e Biotecnologia, PqEB W5 Norte, 70770-900, Brasília, DF, Brazil; 2 Universidade de Brasília, Departamento de Biologia Celular, Campus Universitário, 70910-900, Brasília, DF, Brazil; 3 Instituto Nacional de Ciência e Tecnologia em Interações Planta-Praga, Universidade Federal de Viçosa, 36570.000, Viçosa, MG, Brazil; 4 Universidade Estadual de Maringá, Departamento de Genética e Biologia Celular, Maringá, PR, Brazil

## Abstract

**Background:**

Self-complementary RNA transcripts form a double-stranded RNA (dsRNA) that triggers a sequence-specific mRNA degradation, in a process known as RNA interference (RNAi), leading to gene silencing. In vascular plants, RNAi molecules trafficking occur between cells and systemically throughout the plant. RNAi signals can spread systemically throughout a plant, even across graft junctions from transgenic to non-transgenic stocks. There is also a great interest in applying RNAi to pathogenic fungi. Specific inhibition of gene expression by RNAi has been shown to be suitable for a multitude of phytopathogenic filamentous fungi. However, double-stranded (ds)RNA/small interfering (si)RNA silencing effect has not been observed *in vivo*.

**Results:**

This study demonstrates for the first time the *in vivo *interference phenomenon in the pathogenic fungus *Fusarium verticillioides*, in which expression of an individual fungal transgene was specifically abolished by inoculating mycelial cells in transgenic tobacco plants engineered to express siRNAs from a dsRNA corresponding to the particular transgene.

**Conclusion:**

The results provide a powerful tool for further studies on molecular plant-microbe and symbiotic interactions. From a biotechnological perspective, silencing of fungal genes by generating siRNAs in the host provides a novel strategy for the development of broad fungi-resistance strategies in plants and other organisms.

## Background

The genetic interference phenomenon [RNA interference (RNAi)] was described in *Caenorhabditis elegans *in which double-stranded RNA (dsRNA) induces individual sequence-specific posttranscriptional gene silencing. The spreading silencing effect has been demonstrated, in which the interference is observed in a broad region of the animal after the injection of dsRNA into the extracellular body cavity. Fire *et al*. [1] demonstrated that RNAi abolished expression of targeted genes in *C. elegans *by injecting dsRNA. Subsequently, others have shown that the effect also occurs when *C. elegans *is fed the bacterium *Escherichia coli*, which transcribes the recombinant dsRNA [2]. It also results from simply soaking the animals in dsRNA preparations [3]. Data obtained from *in vivo *and *in vitro *studies are now being used to engineer resistance against parasitic nematodes in transgenic plants [4-6].

Cellular boundaries play a pivotal role in this integration by maintaining a level of cell autonomy while enabling communication between cells for coordinated gene expression and metabolism. In vascular plants, trafficking of RNAi molecules occurs between cells and systemically throughout the plant [7,8]. Such RNA trafficking breaks the boundaries of our traditional thinking of RNAs as functioning solely within the cells in which they are produced, and ushers in a new frontier of plant biology [9]. Intercellular and systemic movement occurs via plasmodesmata, which provide the continuity of cytoplasm and endoplasmic reticulum between adjacent cells and the phloem. Analyses of vascular exudates from oilseed rape (*Brassica napus*) showed that phloem sap contained a large number of small (sm)RNAs, predominantly of 21 and 24 nucleotides in length [10]. In addition, RNAi signals can spread systemically throughout a plant, even across graft junctions from transgenic stocks to non-transgenic scions [11,12]. Moreover, recent experiments described by Tomilov *et al*. [13] demonstrated that the movement of RNAi molecules between a parasite and its host plants. The *gus *silencing signal generated by lettuce roots was functional in its parasite *Triphysaria versicolor*, translocating across the haustorium interface. In nematodes gene silencing may also be triggered by a diet composed of transgene-encoded RNAi plants [4,14]. The same phenomenon has been observed in herbivorous insects fed on a plant engineered to express dsRNAs targeting vital insect genes [15,16].

Plants have been genetically manipulated by introducing constructs encoding self-complementary hairpin RNA (hpRNA) to efficiently silence genes [17-19]. The expressed transcripts form a dsRNA that triggers a sequence-specific messenger (m)RNA degradation (RNAi). Briefly, the dsRNA is recognized by DICER-like enzymes, which cleaves the molecule into a series of 21-23 bp duplexes [called small interfering (si)RNAs], that complexes with a ribonucleoprotein complex (RISC). The duplex is unwound to give single stranded siRNA leading to activation of the RISC, which searches for homologous mRNA transcripts by a base-pairing mechanism, leading to mRNA degradation. In addition, siRNA can affect the chromatin structure of targeted genes, resulting in transcriptional inhibition [20].

There is a great interest in applying RNAi to pathogenic fungi. Specific inhibition of gene expression by RNAi has been shown to be suitable for a multitude of phytopathogenic filamentous fungi, such as *Magnaporthe oryzae *[21], *Sclerotinia sclerotiorum *[22], *Phytophthora sojae *[23], *Aspergillus nidulans *[24], *A. fumigatus *[25-28], *A. oryzae *[29], *Bipolaris oryzae *[30], *Colletotrichum lagenarium *[31], *Coprinus cinereus *[32,33], *Fusarium solani *[34], *Mucor circinelloides *[35], which were transformed with plasmid constructs to express self-complementary hairpin RNA molecules [36]. It has also been shown that simply adding synthetic siRNA molecules to the culture medium can result in specific suppression of the corresponding target gene in *Aspergillus nidulans *[37]. In addition, the reporter *gfp *transgene and the endogenous genes coding for hydrophobins and a peroxiredoxin were silenced in *Moniliophthora perniciosa *transfected with *in vitro *synthesized specific dsRNA [38]. However, the dsRNA/siRNA silencing effect has not been observed *in vivo*. Here we show that the *gus *gene expression can be specifically silenced in *Fusarium verticillioides *(= *F. moniliforme*) interacting with a transgenic plant engineered with a *gus *gene-inferring cassette [hairpin (hp)GUS].

## Results and Discussion

**Figure 1:**
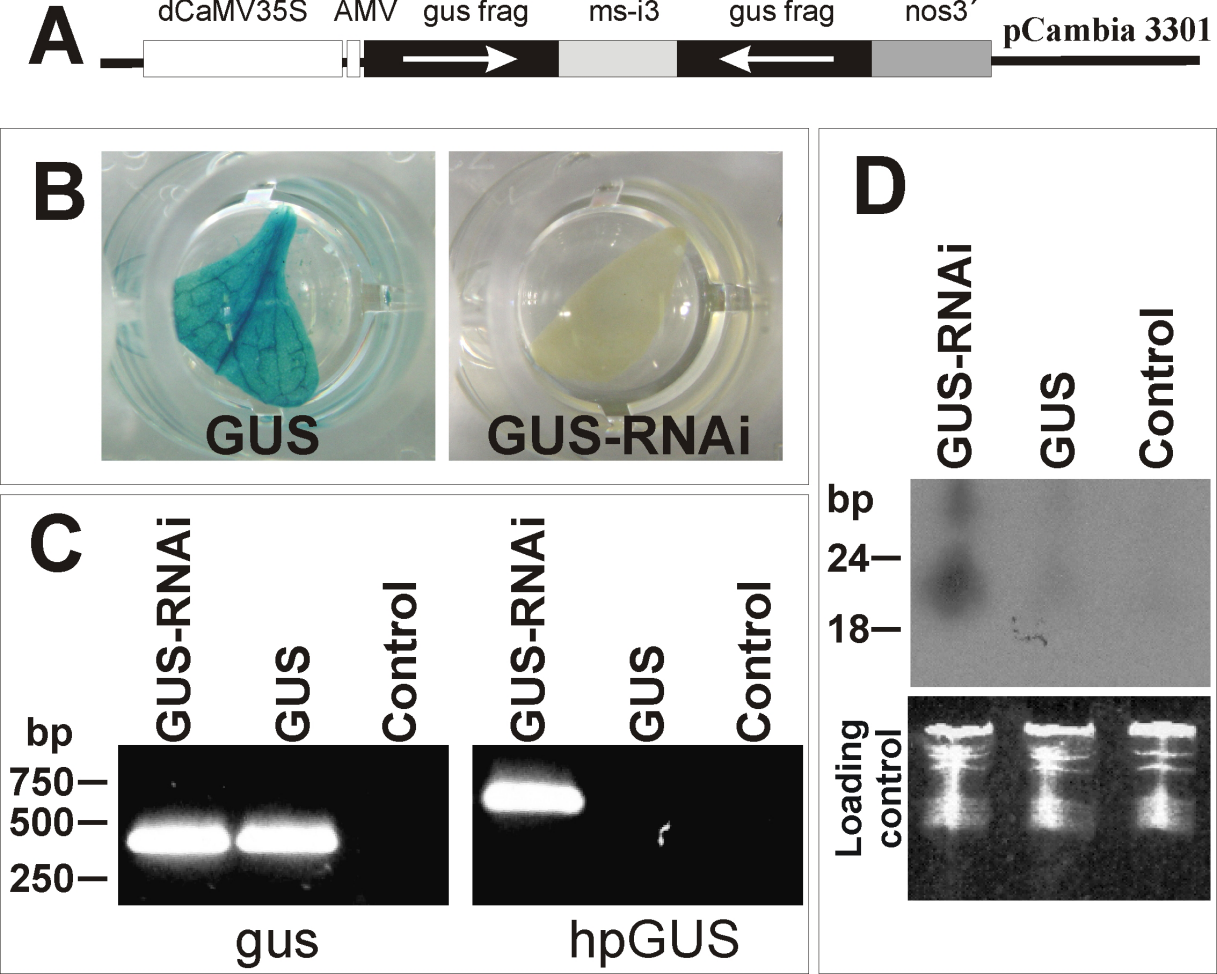
**Tobacco GUS-expressing line was re-transformed to express a double-stranded RNA for silencing the *gus *gene**. (A) General scheme of the intron-spliced hairpin (hp)RNA vector (pC1302GUSi) constructed to promote *gus *gene silencing in GUS^+ ^transformed tobacco lines. In order to generate the *gus *inferring cassette (hpGUS) a 627-bp fragment from *gus *gene (gus frag) was directionally cloned in order to generate sense and antisense arms, flanking the malate synthase gene intron 3 from *Arabidopsis thaliana *(ms-i3). (B) A GUS expressing tobacco line (GUS) was re-transformed with pC1302GUSi and regenerated plants (GUS-RNAi lines) did not show observable *gus *gene expression. (C) transgenic plants were analysed by polymerase chain reaction n order to detect both *gus *transgene in transformed plants (GUS) and the *gus *inferring cassette (hpGUS) in the GUS-RNAi lines. (D) Detection of small interfering RNA in a GUS expressing plants, GUS-RNAi line and control. Ethidium bromide-stained RNA serves as the loading control. Control in C and D is a non-transformed plant. Molecular size markers are indicated on the left in C and D.

In this study, we hypothesized that a plant expressing dsRNA could result in *in vivo *trans-specific suppression of the corresponding target gene in fungal cells attached to plant tissues. Tobacco plants expressing the *gus *gene were re-transformed with an interfering intron-hairpin construct (Figure 1A). None of the re-transformed lines (GUS-RNAi lines) presented an observed *gus *expression in the leaves (Figure 1B) and no significant phenotypical differences were observed in the transgenic lines when compared with wild-type tobacco. Polymerase chain reaction (PCR) analysis confirmed the presence of both transgenes in the GUS-RNAi lines (Figure 1C). Northern analysis was carried out to detect the siRNA in the transgenic tobacco lines showed siRNA bands of expected size range only in the GUS-RNAi lines (Figure 1D). No signal was observed in either *gus*-expressing or non-transgenic plants. The constitutive expression of the *gus *dsRNA in these transgenic tobacco lines provides *gus *siRNA molecules for absorption by *F. verticillioides *feeding cells and subsequent RNAi of the constitutive *gus *transgene in fungal mycelium and conidial cells. In addition, uptake of unprocessed dsRNA and others RNAi molecules could be also occurs.

**Figure 2:**
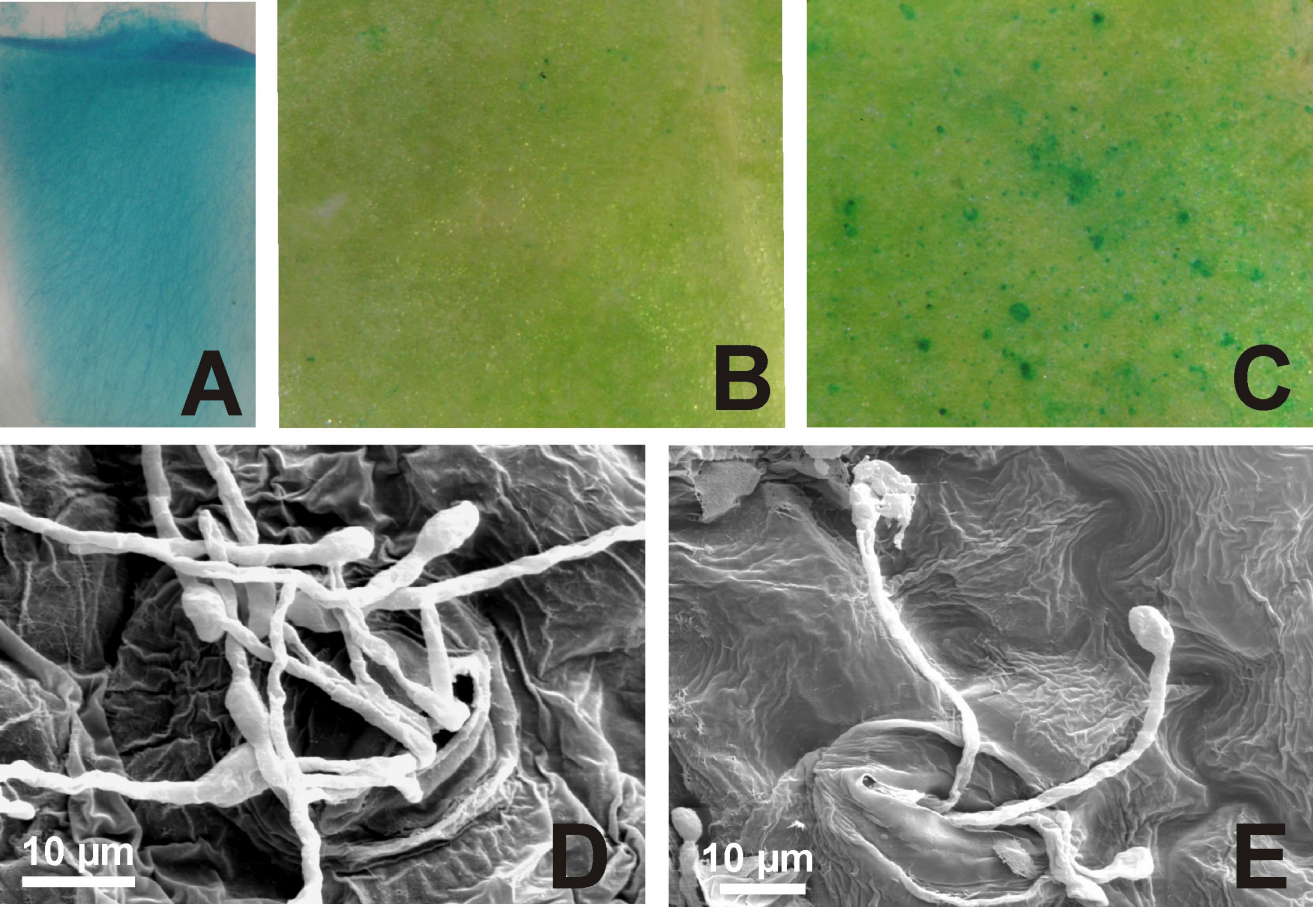
**Inoculations of GUS-RNAi tobacco lines with GUS expressing *Fusarium verticillioides***. (A) Mycelium cells exhibiting stable and high *gus *gene expression were used for the inoculation of plant leaves. (B) GUS assay carried out with GUS-RNAi tobacco leaves inoculated with the GUS^+ ^fungus. Fungal mycelia interacting with the re-transformed plant did not show GUS expression; only a few germinating spores presented GUS expression. (C) GUS assay with non-transformed tobacco leaves inoculated with GUS^+ ^fungus. Fungal mycelia showed high GUS expression level. (D, E) After GUS assay, leaf surfaces (presented in B and C) were observed under a scanning electron microscope in order to observe spores germinating and penetrating into stomata.

*F. verticillioides *fungal strain S68 (a *nia*^-^mutant) was transformed to express the GUS and nitrate reductase coding genes. Only colonies harbouring the nitrate reductase gene (*nia*^+^) were able to grow on MM plates and all co-transformants showed mitotic stability in respect of the GUS^+ ^phenotype. One line over-expressing the *gus *gene was selected to inoculate both GUS-RNAi and non-transformed tobacco lines (Figure 2A). Leaves detached from *in vitro *plants (*n *= 12 per line; repeated twice) were inoculated with *F. verticillioides *culture, applied to the adaxial surface. After 11 days the agar plug was removed and leaves containing penetrative food-absorbing structures that remained attached to leaf surface were analysed for *gus *gene expression. When the *F. verticillioides *expressing the *gus *transgene was inoculated on leaves of GUS-RNAi lines, expressing dsRNA corresponding to the *gus *gene, a small number of fungal structures exhibiting the *gus *gene expression (7 ± 3 blue spots/cm^2^) were observed by GUS protein assay (Figure 2B). Non-transgenic tobacco leaves were unable to cause an evident *gus *gene silencing in transformed fungus structures and a large number of fungal structures exhibiting GUS expression was observed (221 ± 25 blue spots/cm^2^) (Figure 2C). After GUS assay, leaf surfaces were observed under scanning electron microscope. The analysis revealed that only germinating spores remained on the leaf surfaces (Figures 2D-E). Almost all spores observed were penetrating into leaf stomata. These phenotypes convincingly indicate that the silencing signal translocated across the germinated spores from tobacco into fungal cells. Recently, the translocation of RNAi molecules across the haustorium interface was demonstrated between a parasite and its host plants [13]. As a consequence, transgenic lettuce plants expressing a *gus *dsRNA induced specific gene silencing in their parasitic plant *Triphysaria versicolor*.

Infection by *F. verticillioides *can result in highly variable disease symptoms ranging from asymptomatic plants to severe rotting and wilting. In the more virulent pathogen *F. oxysporum *the whole process proceeds much faster and the transition from the initial symptomless phase to the necrotrophic phase occurs within a few days [39]. In contrast, the less aggressive pathogen *F. verticillioides *develops more slowly, leaving the plant more time to respond and restrict fungal growth [40]. In this study, the infection started from fungal conidia that initiated penetration to palisade and spongy mesophyll through stomata ~11 days after inoculation. We speculate that this fungus characteristic could be an important condition for plant-pathogenic fungi interaction-mediated gene silencing. These interaction types establish a long-term feeding relationship with a living host plant cell, allowing the nutrient-absorbing cells to uptake dsRNA molecules from their host. Indeed, this mode of plant colonization was confirmed when early stages of the *F. verticillioides*-maize interaction were characterized by using green fluorescent protein-expressing transgenic fungus isolates. Conidia were found only inside a cell in which an infection was established, while the surrounding cells appeared to be normal [40]. The differences in susceptibility could reflect the resistance of some cells or stages to the consequences of absorbed dsRNA.

In this work, transgene silencing in *F. verticillioides *was achieved by the interaction of fungal cells with a transgenic tobacco engineered for both transcribing and silencing the *gus *transgene expression, allowing the phenomenon of transitive silencing cycles of 'degradative-PCR' to take place in the plant cells. The 'degradative PCR' model proposes that siRNAs act as primers to transform the target mRNA into dsRNA. The nascent dsRNA is degraded by Dicer generating new siRNAs in a circle of dsRNA synthesis and degradation [41,42]. Further investigation should be carried out in order to determine whether this is important. It is known that gene silencing is dose-dependent and that more intact long hpRNAs could cause more pronounced silencing [43].

**Figure 3:**
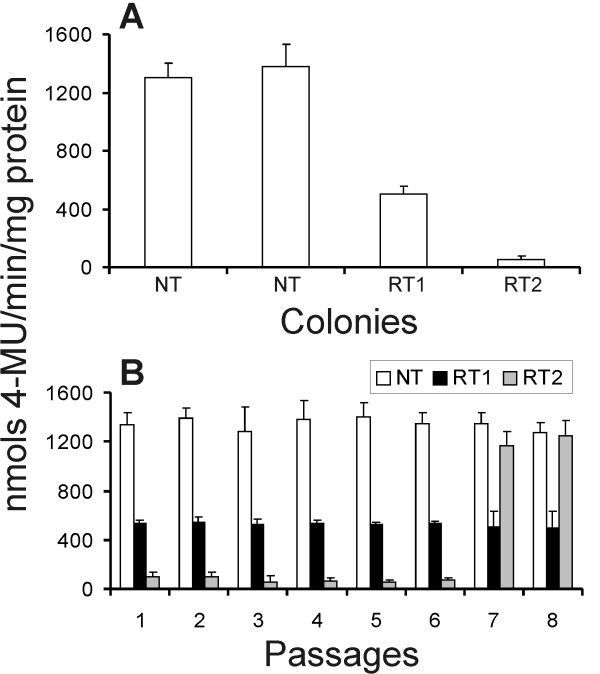
**Isolation of *Fusarium verticillioides *colonies from both re-transformed and non-transformed tobacco lines**. (A) The *gus *gene expression was quantified in fungal colonies isolated from non-transgenic (NT) and transgenic (RT1 and RT2) lines. (B) The two colonies isolated from GUS-RNA interference plant lines presented a reduction of the *gus *gene expression and were analysed for GUS expression over eight passages.

**Figure 4:**
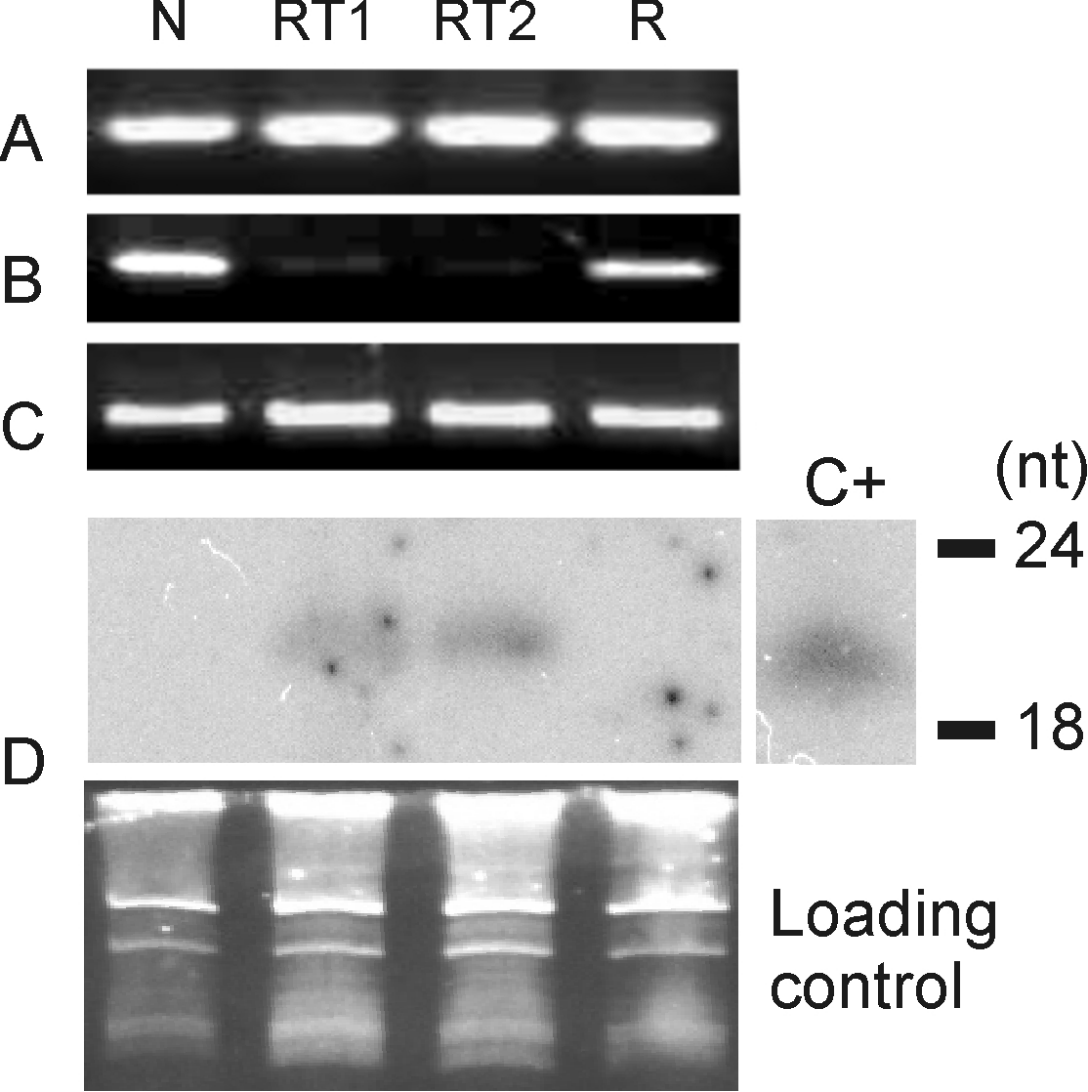
**Presence and expression of the *gus *gene in transformed *Fusarium verticillioides *isolated from *small interfering (*si)RNA-expressing tobacco plants**. (A) Polymerase chain reaction (PCR) analysis for the presence of the *gus *gene in *F. verticillioides *isolates that were not inoculated in tobacco plants (N), which were isolated from inoculated GUS-RNA interference plants and exhibited a reduction of approximately 62% (RT1) and 96% (RT2) in the *gus *gene expression and RT2 isolate after reversion to the normal GUS^+ ^phenotype after the 7^th ^passage (R). (B) Reverse transcriptase (RT)-PCR analysis for the presence of transcripts from the endogenous *gus *gene in fungal cells. (C) RT-PCR analysis for the presence of transcripts from the fungal *5.8S rRNA *housekeeping gene (internal control). (D) Detection of siRNA isolated from fungi isolated from transgenic (RT1, RT2 and R) and non-transgenic plants (N). The position corresponding to 18 and 24 nucleotides is indicated. C+: 100 pg of a *gus *gene-derived oligomer. Ethidium bromide-stained RNA serves as the loading control.

Ten fungal colonies were re-isolated from both GUS-RNAi and non-transformed tobacco lines and established *in vitro*. The *gus *gene expression was quantified in all re-isolated colonies and results revealed that two colonies isolated from GUS-RNAi plant lines presented a reduction of approximately 62% (RT1) and 96% (RT2) in the *gus *gene expression, compared with colonies isolated from non-transformed leaves (Figure 3A). All colonies isolated from non-transformed plants revealed normal GUS expression (activity raging from 1312 to 1378 nmol 4-MU.min^-1^.mg.protein^-1^). We further investigated the stability of gene silencing in mycelia cells over several passages in the absence of the RNAi trigger molecules from GUS-RNAi plants. The colonies showing a reduction of GUS expression were cultured in order to reach 70%-80% of confluence for eight passages (Figure 3B). GUS fluorimetric analyses revealed that the colony that presented 62% GUS expression reduction (RT1) remained expressing the *gus *gene in a similar pattern. However, the colony that presented 96% GUS expression (RT2) resumed normal GUS activity after the seventh passage. Steady-state levels of GUS mRNA in mycelia from the seventh-passage were estimated by semi-quantitative reverse transcription (RT)-PCR. *gus*-specific primers were used to distinguish cDNA from genomic GUS sequences. Results have shown a reduction in the steady-state level of *gus *transcript in colony mycelia of RT1 and RT2, which exhibit partial degrees of transgene silencing. In addition, after GUS-silencing phenotype reversion, the colony RT2 exhibits a *gus *transcript level similar to the control mycelia (which were not attached to hpGUS-tobacco leaves; Figure 4). Moreover, northern analyses showed siRNA bands of expected size range in both RT1 and RT2 silenced colonies and no signal was observed for the fungus colony isolated from non-transformed leaves (N) and RT2 colony that resumed normal GUS activity (R; Figure 4D).

It has been shown that silencing phenotypes induced by RNAi can persist for generations. Despite numerous reports on RNA silencing in a variety of organisms, it is still not understood whether systemic smRNA signalling occurs in filamentous fungi. Nevertheless, silencing was shown to be a reversible dominant trait, operative in heterokaryotic strains containing a mixture of transgenic and non-transgenic nuclei, which suggests that a diffusable, trans-acting mobile RNA signal is involved [38,44]. In the filamentous fungus *M. perniciosa*, the *gfp*-silenced phenotype persisted for a period of 120 days after the transfection with dsRNAs [38]. In contrast, *P. infestans *treated with *gfp*dsRNA exhibited gene expression partially recovered after 4 days [45]. In *Caenorhabditis elegans *a single episode of RNAi in the nematode induced transgene silencing effects that were inherited over 80 generations in the absence of the original trigger [46]. In mice, microinjection of microRNAs into fertilized eggs also induced a heritable silencing phenotype, associated with the zygotic transfer of RNA molecules [47]. In vascular plants, recent studies suggest that smRNAs can move between cells (through plasmodesmata and endoplasmic reticulum) and can spread systemically via the long-distance transport systems (phloem) [48-52]. It was demonstrated that plant siRNAs do not only act at the site of synthesis, but are additionally mobile between cells [53]. There are indications that siRNAs can move 10-15 cells without amplification - probably as molecules of the 21-nt class - whereas movement over greater distances requires an amplification of the original signal [53]. In addition, certain cell types, such as neurons and sperm, are known to be more resistant to RNAi. Athough, so far, no cell type n fungi has been reported to be resistant to RNA silencing, this may possibly be the case with certain fungal cell types. Moreover, we can not exclude the possibility of mutation or an off-target effect on a gene related to silencing mechanism, or paramutation phenomenon associated with the reversion of *gus *gene normal expression.

**Figure 5:**
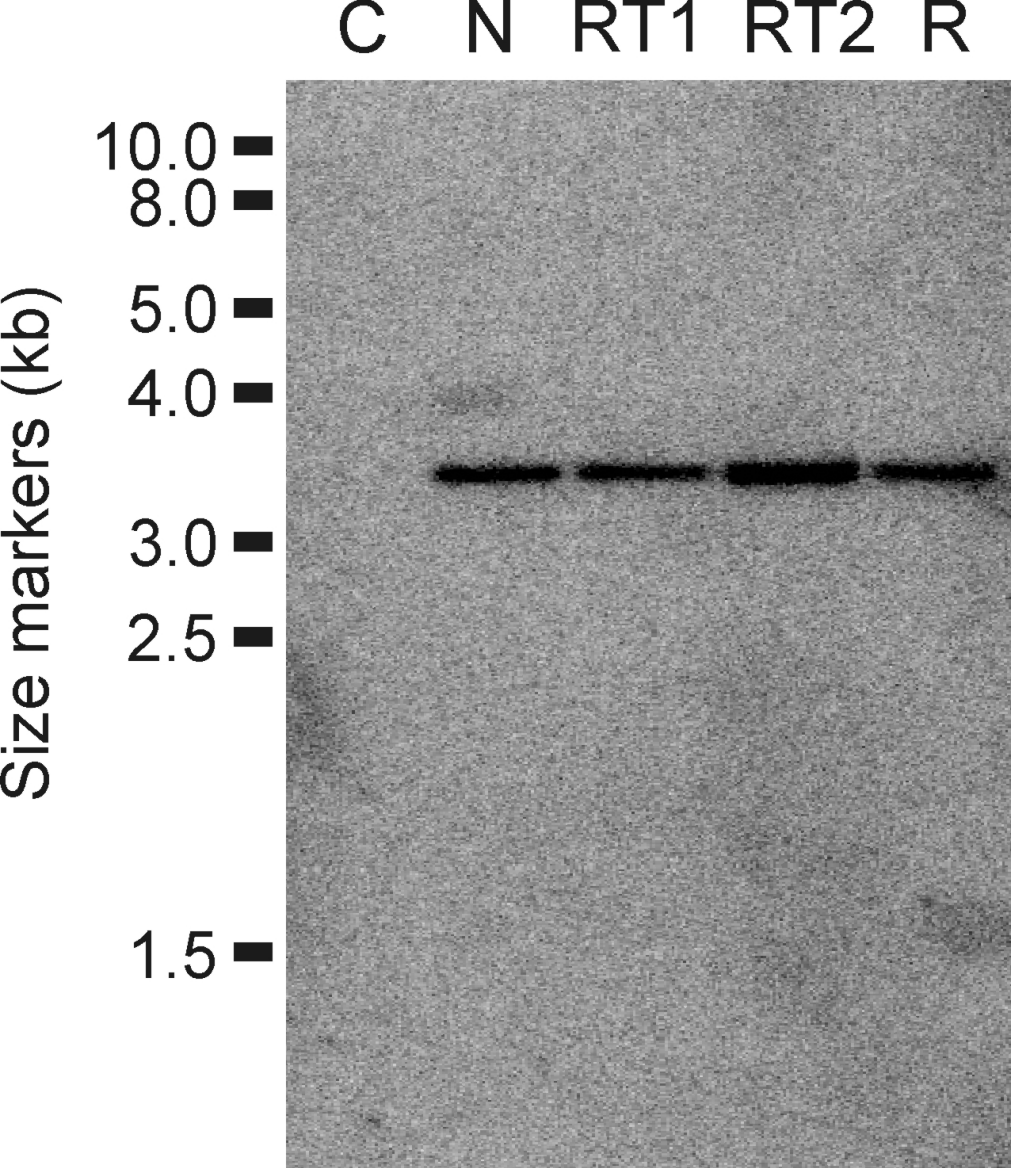
**Southern analysis of genomic DNA to detect the foreign *gus *gene in *Fusarium verticillioides *isolates**. C = non-transformed; N = transgenic isolate that was not inoculated in tobacco plants; RT1 and RT2 = isolated from inoculated GUS-RNA interference plants; R = RT2 isolate after reversion to the normal GUS^+ ^phenotype.

In fungi, transgene gene silencing or instability of the silencing effect has been observed to associated with modifications in the organization of the transgene loci [31,44,54-56]. In order to investigate whether the *gus *gene silencing in fungal cells would be associated to changes in the status of the transgene in the genome, Southern analyses were carried out with fungal colonies that were re-isolated from both GUS-RNAi and non-transformed tobacco lines. Results have shown that all isolates presented a simple and unaffected integration pattern (Figure 5), demonstrating that the transgene locus was stably maintained in the fungal colonies re-isolated from GUS-RNAi plants and after several cultivation passages.

## Conclusions

This work provides an example of RNA-mediated transfer of information between organisms and between plant and pathogen. Further studies should be carried out in order to access whether such RNA-mediated interference-transfer mechanisms participate in natural interactions. The use of siRNAs (RNAi) has become a powerful tool for specifically down-regulating gene expression and it has been demonstrated to be of great importance in basic and applied molecular plant-pathogen, mixotrophic and symbiotic interaction studies. From a biotechnological perspective, our results of *in planta *RNAi silencing of a gene expressed in a parasite fungus open new perspectives for the development of broad fungi-resistance strategies in plants and other organisms.

## Methods

### Plasmid construct and transgenic plants

Tobacco (cv Xanthi) was transformed as previously described by Horch *et al*. [57] with the vector pBI121 (Clontech) to express the *gus *gene. Transgenic lines presenting high level *gus *gene expression were re-transformed with the vector pC1302GUSi (Figure 1A). pC1302GUSi is an intron-spliced hpRNA vector constructed to promote *gus *gene silencing in re-transformed lines. In order to construct the pC1302GUSi, intron 3 from the malate synthase gene from *Arabidopsis thaliana *(ms-i3; GenBank accession number AB005235) was PCR-amplified from genomic DNA by using Platinum Taq DNA Polymerase High Fidelity (Invitrogen, CA, USA) and specific primers (5'-CTCTAGAGGCGCGCCGGTACCCCGGATCCATCAGCCTGTTCAAA-3' and 5'-TGAGCTCTCGCGATGGGGCCCACTAGTTTTATGGTCCATTTTC-3'), generating sites for *Xba*I, *Asc*I, *Kpn*I, *Bam*HI and *Spe*I, *Apa*I, *Nru*I, *Sac*I, respectively. The 438-pb fragment was cloned into pCR2.1-TOPO (Invitrogen) and sequenced. The ms-i3 fragment was then removed with *Sac*I and *Xba*I and inserted into the vector pUC19-35SAMVNOS [58] in order to generate the vector pSIU. A 627-bp fragment from *gus *gene coding sequence was removed with *Ssp*I and *Eco*RV from the vector pCambia3301 (Cambia, Brisbane, Australia) and cloned into the *Sma*I site from the pBlueScript KS+ (Fermentas, Ontario, Canada). The *gus *fragment was finally directionally cloned into the sites *Nru*I and *Kpn*I/*Xba*I from pSIU in order to generate sense and antisense arms, flanking the malate synthase gene intron 3 from *Arabidopsis thaliana *(GenBank accession No. AB005235). The *gus *inferring cassette (hpGUS) was removed with *Pvu*II and cloned into *Sma*I from pCambia1302 (Cambia) generating the vector pC1302GUSi.

PCR analyses were carried out in order to detect the primary *gus *gene and the silencing cassette in the transformed and re-transformed (GUS-RNAi) lines. The primer pair GUS251 (5'-TTGGGCAGGCCAGCGATACGT-3') and GUS671 (5'-ATAACGCAGTTCAACGCTGAC-3') was used to amplify 420 bp from the functional *gus *gene and the primer pair GUSF (5'-TCAGGAAGTGATGGAGCATCAGG-3') and MSIR (5'-TAGTTGGTCTGGCAGGCTAGT-3') were used to amplify 627 bp from intro-spliced hpRNA vector (pC1302GUSi). PCR amplifications contained 0.4 μM of primer, 250 μM dNTP's, 1.5 μM MgCl2, 1.0 U Taq DNA polymerase (5 U/μL) and about 20 ng of genomic DNA. Temperature cycling was performed on a PCT-100 thermocycler (MJ Research, MA, USA) as follows: initial denaturation at 95°C for 5 min; 36 cycles of denaturation at 95°C for 1 min; primer annealing at 55°C for 1 min; extension at 72°C for 1 min; and a final extension at 72°C for 5 min.

### Transgenic Fusarium

*F. verticillioides *strain S68 (a *nia*^-^mutant) was previously co-transformed with the vectors pNOM102 containing the *gus *gene under control of the *gpd *promoter from *A. nidulans*, and pNH24 carrying the *F. oxysporum *nitrate reductase gene (*nia*). The line TG8 exhibiting the *nia*^+^phenotype conferred by pNH24 was selected on MM with nitrate as the sole nitrogen source and purified by isolation of uninucleated conidia according to standard techniques. Only colonies harbouring the pNH24 vector (*nia*^+^) are able to grow on MM plates. The line TG8 was previously characterized and presented a single copy of the *gus *gene integrated into the genome and mitotic stability in respect of the GUS^+ ^phenotype [59].

### Inoculation of transgenic plants with gus^+ ^Fusarium

One transgenic line of *F. verticillioides *over-expressing the *gus *gene was selected to inoculate both re-transformed and non-transformed tobacco lines. The fungus was grown on MM medium [59] at 26°C. Inoculation was carried out according to Dias *et al*. [58]. A mycelial agar plug 3 mm in diameter was cut from the growing margins of a 6-day-old *F. verticillioides *culture and applied to the adaxial surface of a leaf detached from 6-week-old *in vitro *plants (*n *= 12 per line; repeated twice). After 11 days at 26°C and under 90%-100% relative humidity, the agar plug was removed and leaves containing penetrative food-absorbing structures that remained attached to leaf surface were analysed for *gus *gene expression.

Fungal colonies were re-isolated from both GUS-RNAi and non-transformed tobacco lines. Small portions from mycelium were removed under stereomicroscope and cultured on MM medium (as described above) in order to reach 70%-80% of confluence for eight passages. GUS histochemical and fluorimetric assays were carried out in each passage. The qualitative β-glucuronidase activity assay was carried out according to Couteaudier *et al*. [60] using 4-methyl umbeliferyl β-D-glucuronide as a substrate. Fluorescence was measured on a TKO100 Minifluorimeter (Hoefer, CA, USA) at 365_nm_. Protein content was determined using a protein assay kit (BioRad, CA, USA).

### GUS assays and scanning electron microscopy

The GUS histochemical assay was performed as described by Jefferson [61]. Stained materials were examined using a Zeiss Axiophot stereomicroscope and photographed using a Zeiss AxioCam ICC3 high-resolution digital camera system. Explants were also examined using a scanning microscope. For scanning microscopy, leaves were fixed with 3% glutaldeihyde in 0.1 M sodium cacodylate buffer at pH 7.2 for 15 h at 4°C and postfixed in 1% osmium tetroxide for 1 h. After washing in the same buffer, the specimens were dehydrated in a graded acetone series, critical-point dried with CO2, sputter-coated with a thin layer of gold and observed under a Zeiss DSM 962 SEM operating at 15 kV.

### RT-PCR expression analysis

Mycelium cells were used for total RNA extraction as described, with Trizol (Invitrogen) as recommended by the manufacturer. Total RNA was used to produce cDNA using the reverse transcriptase Superscript III (Invitrogen), according to the protocol suggested by the manufacturer. PCRs were carried out as described [62], except that 20 ng of cDNA was used as a template, in reactions with 32 cycles of amplification. The number of amplification cycles was previously optimized in order to stop the reaction at the exponential stage, ensuring that amplification was semi-quantitative. Primers 5'-ATCACGCAGTTCAACGCTGAC-3' (GUS671) and 5'-TTGGGCAGGCCAGCGTATCGT-3' (GUS251) were used to amplify a 420 bp fragment from the *gus *gene. As an internal control, primers 5'-GGAAGTAAAAGTCGTAACAAGG-3' (ITS5) and 5'-TCCTCCGCTTATTGATATGC-3' (ITS4) were utilized to amplify a fragment of the *5.8S rRNA *housekeeping gene. PCRs with total RNA presented no amplified fragments. Experiments were repeated three times.

### RNAi analysis

The mycelia of the fungi were cultivated on static liquid MM medium for 14 days at 26°C. Total RNA was isolated by extraction with Micro-to-Midi Total RNA Purification System (Invitrogen) as recommended by the manufacturer. Total RNA from plant leaves was isolated according to Bonfim *et al*. [19]. siRNA analysis was carried out according to Bonfim *et al*. [19]. RNAs were hybridized with a DNA probe corresponding to the 627 pb-fragment from *gus *gene, excised with *Eco*RV and *Ssp*I from pCambia2301. Probes were labelled with α^32^P dCTP using a random primer DNA labelling kit (Amersham Pharmacia Biotech, NJ, USA) according to the manufacturer's instructions. Three oligomers (18, 24 and 38 nucleotides) were used as molecular size markers. A GUS-sequence-related 21-nucleotide oligomer was used as a positive control. The bands were visualized with a fluorescent image analyser (FLA-3000) (FUJIFILM, Tokyo, Japan).

### Southern blot analysis

Genomic DNA was isolated as described by Chow *et al*. [63]. Southern blotting was carried out as described [64]. Genomic DNA (20 μg) was digested with *Sph*I, separated on a 1% agarose gel, and transferred to a nylon membrane (Hybond-N+; Amersham Pharmacia Biotech). Hybridizations were carried out using the PCR amplified 420-bp fragment from the *gus *gene, labelled with α^32^P deoxycytidine triphosphate (3000 Ci mol^-1^) using a random primer DNA-labelling kit (Amersham Pharmacia Biotech) according to the manufacturer's instructions. The bands were visualized with a fluorescent image analyser (FLA-3000; FUJIFILM).

## Abbreviations

dsRNA: double-stranded RNA; hpGUS: hairpin GUS; hpRNA: hairpin RNA; mRNA: messenger RNA; PCR: polymerase chain reaction; RISC: ribonucleoprotein complex; RNAi: RNA interference; RT-PCR: reverse transcription PCR; siRNA: small interfering RNA; smRNA: small RNA.

## Authors' contributions

MLPT carried out the molecular studies and *in vivo *assays, analysed the data and drafted the manuscript. BBAD carried out the fungi inoculation assays. JAP performed fungi transformation. RCDA participated in the molecular analysis. FJLA is the research group leader, conceived the study and participated in its design, analysed the data and finalized the manuscript. All authors read and approved the final manuscript.
